# Low Calorie Beverage Consumption Is Associated with Energy and Nutrient Intakes and Diet Quality in British Adults

**DOI:** 10.3390/nu8010009

**Published:** 2016-01-02

**Authors:** Sigrid A. Gibson, Graham W. Horgan, Lucy E. Francis, Amelia A. Gibson, Alison M. Stephen

**Affiliations:** 1 Sig-Nurture, Guildford, Surrey GU1 2TF, UK; lucyefrancis@gmail.com (L.E.F.); amelia@sig-nurture.com (A.A.G.); 2 Biomathematics and Statistics Scotland, Aberdeen AB21 9SB, Scotland; g.horgan@abdn.ac.uk; 3 Department of Nutritional Sciences, University of Surrey, Guildford, Surrey GU2 7XH, UK; amlennox@btinternet.com

**Keywords:** low calorie, beverage, diet, energy intake, nutrient intake, soft drinks

## Abstract

It is unclear whether consumption of low-calorie beverages (LCB) leads to compensatory consumption of sweet foods, thus reducing benefits for weight control or diet quality. This analysis investigated associations between beverage consumption and energy intake and diet quality of adults in the UK National Diet and Nutrition Survey (NDNS) (2008–2011; *n* = 1590), classified into: (a) non-consumers of soft drinks (NC); (b) LCB consumers; (c) sugar-sweetened beverage (SSB) consumers; or (d) consumers of both beverages (BB), based on 4-day dietary records. Within-person data on beverage consumption on different days assessed the impact on energy intake. LCB consumers and NC consumed less energy and non-milk extrinsic sugars than other groups. Micronutrient intakes and food choices suggested higher dietary quality in NC/LCB consumers compared with SSB/BB consumers. Within individuals on different days, consumption of SSB, milk, juice, and alcohol were all associated with increased energy intake, while LCB and tea, coffee or water were associated with no change; or reduced energy intake when substituted for caloric beverages. Results indicate that NC and LCB consumers tend to have higher quality diets compared with SSB or BB consumers and do not compensate for sugar or energy deficits by consuming more sugary foods.

## 1. Introduction

In the latest results from the UK National Diet and Nutrition Survey [1], 44% by weight of all soft drinks consumed by adults aged 19–64 years were low-calorie beverages (LCB), a higher proportion than in other European countries [2]. In the United States, where per capita consumption of beverages is roughly double that in the UK [3], results of NHANES 2003–2010 showed that LCB constituted 32% of beverages among adults and 19% among children [4]. As a substitute for sugar-sweetened beverages (SSB), LCB offer the potential to satisfy both thirst and an innate desire for sweetness [5] with minimal caloric load [6]. Replacing an energy-containing beverage with an energy-free one may reduce energy intake, depending on the extent of compensation, both short-term and long-term [7,8,9]. The majority of randomised controlled trials (RCTs) among adults suggest that using LCB instead of caloric beverages over several weeks or months results in modest weight loss [10,11,12,13], although results vary by age, sex, ethnicity, or weight status groups [14]. Trials in overweight adolescents [15] and normal weight children [16] have found that replacing SSB with drinks like water or LCB may prevent weight gain. One adult weight loss trial found greater weight loss with LCB compared to water [17] and a recent systematic review and meta-analysis of ad libitum studies also concluded that use of low energy sweeteners (LCS) in place of sugar leads to reduced energy intake (EI) and bodyweight, possibly also when compared with water [18].

While most expert committees advise reduced consumption of SSB to reduce energy intake [19,20], they vary in advice about substituting with LCB. One reason may be a fear that LCB may encourage compensatory overeating of sweet foods or foods lower in nutrients. The impact on dietary quality should be included in assessing the overall balance of benefits and risks for LCS and LCB [21], but data are limited. In a review of the impact of LCS on weight management, Anderson *et al.* found that users of LCB or LCS reported higher quality diets than non-users [22], although not all studies were consistent. Using NHANES data from 1999 to 2008, Drewnowski and Rehm found that consumers of LCS, were more likely to be female, white, older, and of higher socio-economic status, to be non-smokers and more physically active than non-consumers of LCS, suggesting that LCS consumption is a marker for a healthy diet and lifestyle [23]. Moreover, users of LCB as well as LCS had significantly higher quality diets than non-users.

The effect of LCB on diet quality will depend on the way such beverages are used in the context of the overall diet. In order to target policy and advice appropriately there is a need to explore the dietary composition of those who drink LCB, SSB or no soft drinks. The NDNS rolling programme, with recent data from a representative sample of the UK population, provides an opportunity to conduct such a study. A novel aspect of our paper is the use of 4 days of data for within-person analysis of beverage consumption and energy intake.

## 2. Methods

### 2.1. Sample

The NDNS is the most authoritative source of quantitative information on the food habits and nutrient intake of the UK population. Jointly funded by the Department of Health in England (now Public Health England) and the Food Standards Agency, the results are used by Government to develop policy and monitor trends in diet and nutrient intakes [1]. Data files from 3 years (2008–2011) of the NDNS Rolling Programme [24] were obtained under license from the UK Data Archive (http://www.esds.ac.uk). Households were sampled from the UK Postcode Address File, with one adult and one child (18 months or older), or one child selected for inclusion.

### 2.2. Interview

Participants completed a detailed computer assisted personal interview (CAPI) to obtain background information (age, gender, ethnicity, region) and eating and lifestyle behaviours such as smoking, dieting to lose weight, medication and supplement use. Education was classified into eight categories, consolidated into two groups for analysis (any qualifications *vs.* none). Participants were also classified by employment status: full or part-time employment, school or college full-time, or not working at present. Their National Statistics Socio-Economic Classification (NS-SEC) was determined, based on occupation and degree of supervisory responsibility (8 categories), consolidated into four categories for analysis: (a) Higher professional/managerial; (b) Lower professional/managerial; (c) Intermediate/small employer; and (d) Routine/manual/not working. Information was also collected on household income band.

### 2.3. Diet Record

Following the questionnaire respondents were asked to complete a dietary record for 4 consecutive days, giving a detailed description of each item consumed, time of consumption, and amount, using household measures and photographs. Repeat visits were made by interviewers to check records and probe for missing items. Fifty-five percent of those eligible to participate completed at least 3 days of the 4 days of dietary assessment. Anthropometric measurements (weight, height, waist circumference), taken by trained nurses, were obtained for 90% of those completing a diet record. Trained diet coders entered the food intake data from completed records using an in-house dietary assessment system at MRC Human Nutrition Research, DINO (Data In, Nutrients Out) [25] and using a databank of more than 7000 foods, regularly updated and extended for the survey. Volumes of tea, coffee and concentrated soft drinks included water added as diluent. In NDNS files, tea and coffee excluded contribution from milk and sugar (except for premixed tea and coffee from takeaway/vending sources). Milk was listed separately, while discretionary sugar was treated as a food. Nutrients were calculated using the food composition databank. Sugars (nutrient) are defined in NDNS as total sugars and also as *non-milk extrinsic sugars*; the latter includes all sugars added by the consumer or in processing plus sugars in fruit juice and 50% of sugars in dried and processed fruit.

### 2.4. Classification of Beverages

Beverages were classified as:

1. Low calorie beverages (LCB): all low- and no-calorie beverages, carbonated and still, ready to drink and diluted (weight of added water included). Excludes water.
2. Sugar-sweetened beverages (SSB): beverages with a range of sugar contents, carbonated and still, ready to drink and diluted (weight of added water included).
3. 100% fruit juices (FJ), not including fruit drinks.
4. Tea, coffee and water (TCW), excluding added milk (unless takeaway/vending beverage).
5. Milk: all liquid milk, including that added to hot beverages.
6. Alcoholic beverages: beer, cider, wine, spirits, alco-pops.

### 2.5. Classification of Respondents

Adults aged 16 years and over were classified into four groups according to whether or not they consumed LCB, SSB, both types (BB) or neither (NC) at any time over the 4 days of survey, as shown in Table 1.

**Table 1:** Classification of consumers according to consumption of Low Calorie Beverages (LCB) and Sugar-sweetened Beverages (SSB).

	No LCB	LCB	Total *N*
No SSB	Non-consumer (NC)	LCB only	
	598	216	814
SSB	SSB only	Both LCB and SSB (BB)	
	476	300	776
Total N	1074	516	1590

### 2.6. Statistical Methods

Differences in food and nutrient intake between beverage consumer groups were evaluated using ANOVA and multiple comparison tests with Bonferroni correction. In addition, planned contrasts (based on the null hypothesis that diets of LCB consumers did not differ from others) were used to compare LCB with each of the other groups for greater power. Homogeneity of variances between beverage groups was assessed using Levene’s test. Linear models were used to estimate the mean difference in EI between beverage categories, adjusted for covariates (age, sex, BMI status, ethnicity, education, social class, income, smoking and dieting practice). Weighting (NDNS variable: wti_adY12316) was used to adjust for sampling and non-response bias. Variation within individuals between different days (*n* = 6328 days) was studied using fixed effects models with individual identity, day of the week and day number (day 1 to days 4) as fixed effects to evaluate the impact on EI of varying consumption of LCB from day to day, using each individual as their own control. Beverages were entered as linear covariates (g/day). Two types of models were explored: (1) an unconstrained model of the impact of each beverage on EI if all other beverages and foods were allowed to vary as they do in practice; and (2) a substitution model of the effect of replacing LCB with other beverages. All data were analysed using SPSS version22 (IBM Statistics Inc., Portsmouth, Hants, UK).

## 3. Results

### 3.1. Subject Characteristics

Non-consumers (NC) were older than other groups, BB were youngest and there was no difference in mean age between LCB consumers and SSB consumers (Table 2). There were similar proportions of men and women in each beverage group. LCB consumers were more likely to be white (Caucasian) than SSB consumers. NC were least likely to have formal educational qualifications and were more likely to be non-working/retired. There was no significant difference in socio-economic classification overall, although a slightly higher proportion of LCB consumers had higher professional and managerial occupations, compared with those consuming both LCB and SSB (BB). Similarly, there was no significant difference in household income classification but a slightly higher proportion of LCB consumers had a household income over £30K compared to SSB consumers. In regard to health behaviours reported at interview, current smoking habits did not differ but LCB consumers and NC were more likely to be ex-smokers (*p* = 0.001), and LCB consumers were most likely to drink some alcohol (*p* = 0.03). There was no significant difference in the prevalence of dieting between groups. LCB consumers had a higher mean BMI compared with SSB consumers (mean 28.4 *vs.* 26.3 kg/m^2^; *p* < 0.0001) and were more likely to be obese (33% *vs.* 22%) (*p* = 0.001).

**Table 2:** Background characteristics of beverage consumer groups.

Characteristic	Group		Beverage Consumer Group ^1^					Chi-Square *p* Value
			NC	LCB	SSB	BB	All	
	*n*		598	216	476	300	1590	
Age	All 16+	mean	55 ^a^	46 ^b^	43 ^b^	37 ^c^	47	<0.0001
		SE	1	1	1	1		
Age group	16–24	%	5 ^a^	6 ^a^	24 ^b^	27 ^b^	15	<0.0001
	25–44	%	25	43	33	46	34	
	45–64	%	38	39	27	21	31	
	65+	%	33	13	16	7	20	
Sex	Male	%	47	48	52	47	49	0.25
	Female	%	53	53	48	53	51	
Ethnic group	White	%	89	96 ^a^	86 ^b^	91	89	0.002
	Non-white	%	11	4	14	9	11	
Socio-economic group	Higher Professional‎‎/managerial	%	16	20 ^a^	14	11 ^b^	15	0.26
	Lower Professional/managerial	%	27	27	28	30	28	
	Intermediate/small employers	%	20	17	22	18	20	
	Routine/manual/not working	%	38	36	37	41	38	
Educational Qualifications ^2^	Some	%	72 ^a^	84 ^b^	78	85 ^b^	78	<0.001
	None	%	28	16	22	15	22	
Economic status	In full-time education	%	3 ^a^	2 ^a^	10 ^b^	15 ^b^	7	<0.0001
	Employed	%	51 ^a^	66 ^b^	55	62 ^b^	56	
	Not working or retired	%	47 ^a^	32 ^b^	35 ^b^	23 ^c^	37	
Household income (£)	<15 K	%	17	15	18	13	16	0.08
	15–30 K	%	34	27	37	32	34	
	>30 K	%	50 ^a,b^	58 ^a^	45 ^b^	55 ^a,b^	50	
On a diet	Not dieting	%	87	85	91	88	88	0.11
	Dieting	%	13	15	9	12	12	
Smoking status	Current smoker	%	19	25	23	23	22	0.001
	Ex-regular smoker	%	27 ^a^	27 ^a^	19 ^b^	16 ^b^	22	
	Never regular smoker	%	55	49	58	61	56	
Drink alcohol	Yes	%	81	89	82	86	83	0.03
	No	%	20	12	18	14	17	
BMI group	Under 18.5 kg/m^2^	%	1	1	4	0	2	<0.001
	18.5 and below 25 kg/m^2^	%	33	28 ^a^	39 ^b^	42 ^b^	36	
	25 and below 30 kg/m^2^	%	37	38	34	32	35	
	30+ kg/m^2^	%	28	33 ^a^	22 ^b^	26	27	
BMI (kg/m^2^)		Mean	27.7 ^a^	28.4 ^a^	26.3 ^b^	27.2	27.2	0.001
		SE	0.24	0.4	0.24	0.34	0.14	

^1^ NC, non-consumer; LCB, Low Calorie Beverages; SSB, Sugar Sweetened Beverages; BB, both LCB and SSB ^2^ Educational qualifications: Any qualifications (degree/A levels/GCSE/foreign qualification/still in fulltime education) *vs.* no qualifications. Values sharing the same postscript (or none) are not significantly different (*T*-test, or *Z*-test of column proportions with Bonferroni correction for multiple comparisons).

### 3.2. Beverage Consumption

Mean consumption of LCB was 297 g/day (LCB group) and of SSB 253 g/day (SSB group), while consumers of both types drank on average 548 g/day, with almost equal amounts of LCB and SSB (Table 3). Total fluid intake from beverages (*i.e.*, excluding water in foods) was slightly lower among NC compared with other groups (mean 1679 g/day *vs.* >1800 g/day; *p* = 0.008). NC drank more tea than SSB and BB, and less alcohol than SSB. There was no significant difference in the consumption of milk, fruit juice or plain water between the four groups.

**Table 3:** Beverage consumption (g/day) according to beverage consumer group.

Beverage		Beverage Consumer Group					Overall ANOVA	Planned Contrasts		
		NC	LCB	SSB	BB	All		LCB *vs.* NC	LCB *vs.* SSB	LCB *vs.* BB
	*n*	598	216	476	300	1590				
Sugar-sweetened beverages (SSB)	Mean	0 ^a^	0 ^a^	253 ^b^	263 ^b^	129	<0.0001	1.0	<0.0001	<0.0001
	SE	0	0	11	17	6				
Low calorie beverages (LCB)	Mean	0 ^a^	297 ^b^	0 ^a^	284 ^b^	90	<0.0001	<0.0001	<0.0001	0.67
	SE	0	24	0	18	6				
Tea, coffee, water	Mean	1245 ^a^	1070 ^b^	1041 ^b^	821 ^c^	1080	<0.0001	0.001	0.6	<0.0001
	SE	25	44	29	30	16				
Tea	Mean	490 ^a^	423 ^a,b^	359 ^b,c^	270 ^c^	399	<0.0001	0.05	0.06	<0.0001
	SE	18	29	19	18	10				
Coffee	Mean	281 ^a^	256 ^a^	238 ^a,b^	196 ^b^	249	0.005	0.4	0.50	0.04
	SE	15	22	16	18	9				
Tap Water	Mean	342	310	348	290	330	0.2	0.3	0.3	0.7
	SE	19	31	23	22	12				
Bottled Water	Mean	98	71	78	56	80	0.05	0.1	0.6	0.3
	SE	12	12	8	8	6				
Fruit Juice	Mean	48 ^a^	58 ^a,b^	56 ^b^	62 ^b^	55	0.3	0.3	0.9	0.8
	SE	4	9	4	9	3				
Alcoholic drinks	Mean	222 ^a^	219 ^a,b^	316 ^b^	268 ^a,b^	260	0.02	0.9	0.04	0.3
	SE	16	36	30	33	13				
Milk	Mean	164	163	154	152	158	0.5	1.0	0.5	0.45
	SE	5	11	6	9	3				
Total Beverages	Mean	1679 ^a^	1807 ^a,b^	1821 ^a,b^	1850 ^b^	1772	0.008	0.05	0.85	0.6
	SE	30	57	42	53	21				

Values sharing the same postscript or none are not significantly different (multiple comparison *t*-test with Bonferroni correction). Planned contrasts of LCB *vs.* each of the other beverages as specified *a priori*. Tests assume equal/unequal variance based on Levene’s test.

### 3.3. Food Consumption

In terms of food choices, NC appeared to have the most health conscious diets and BB the least (Table 4). NC ate significantly more fruit and vegetables and fish than SSB or BB, while LCB consumers had similar intakes to NC, and ate significantly more fruit, vegetables and fish than BB. BB consumers ate significantly more meat and meat products, chips and white bread than either LCB or NC. NC ate more high-fibre breakfast cereal and less confectionery than all other groups. There were fewer significant differences between LCB and SSB consumers although LCB consumers ate less sugar and jam than SSB. However the total amount of processed sugary foods, excluding drinks, (*i.e.*, biscuits, cakes, puddings and ice cream, confectionery, sugar and sweet spreads) was similar across all beverage groups (mean: 72, 71, 79, 75 g/day in NC, LCB consumers, SSB consumers, BB consumers, respectively; *p* = 0.37).

**Table 4:** Food consumption (g/day) according to beverage consumer group.

Food Group		Beverage Consumer Group					Planned Contrasts			
		NC	LCB	SSB	BB	All	Overall ANOVA	LCB *vs.* NC	LCB *vs.* SSB	LCB *vs.* BB
	*n*	598	216	476	300	1590				
Meat and Meat Products	Mean	167 ^a^	181 ^a,b^	193 ^b,c^	211 ^c^	185	<0.0001	0.1	0.2	0.009
	SE	5	8	5	8	3				
Fish	Mean	46 ^a^	42 ^a,b^	34 ^b^	31 ^b,c^	39	<0.0001	0.4	0.09	0.03
	SE	2.4	3.9	2.1	2.7	1.3				
Fruit	Mean	110 ^a^	105 ^a,b^	88 ^b,c^	74 ^c^	96	<0.0001	0.6	0.05	0.001
	SE	5	7	5	5	3				
Vegetables	Mean	147 ^a^	137 ^a,b^	129 ^b,c^	112 ^c^	133	<0.0001	0.2	0.3	0.005
	SE	4	7	4	5	3				
Potatoes	Mean	84 ^a^	82 ^a^	88 ^a,b^	99 ^b^	88	0.004	0.8	0.3	0.004
	SE	3	4	3	4	2				
Chips, Fried and Roast Potatoes	Mean	35 ^a^	38 ^a,b^	45 ^b,c^	52 ^c^	42	<0.0001	0.4	0.1	0.004
	SE	2	3	2	3	1				
Bread	Mean	80 ^a^	80 ^a^	88 ^a,b^	90 ^b^	84	0.006	1.0	0.05	0.04
	SE	2	3	2	3	1				
*White bread*	Mean	43 ^a^	42 ^a,b^	54 ^b,c^	58 ^c^	49	<0.0001	0.8	0.002	0.001
	SE	2	2	1	2	1				
Pasta, Rice & other cereals	Mean	65 ^a^	65 ^a,b^	80 ^b^	88 ^b,c^	74	<0.0001	1.0	0.02	0.002
	SE	3	5	4	5	2				
Breakfast cereals	Mean	34 ^a^	23 ^b^	26 ^b^	20 ^b^	28	<0.0001	0.001	0.3	0.3
	SE	2	2	2	1	1				
*High fibre breakfast cereals*	Mean	29 ^a^	19 ^a,b^	19 ^b^	13 ^b,c^	21	<0.0001	0.002	0.8	0.04
	SE	2	2	2	1	1				
Sugary foods	Mean	73	71	78	73	74	0.4	0.7	0.1	0.7
	SE	3	4	3	3	2				
*Puddings, yogurt and ice cream*	Mean	50	48	49	45	49	0.9	0.7	0.8	0.6
	SE	3	4	3	3	2				
*Biscuits and cakes*	Mean	34	32	33	31	33	0.7	0.5	0.7	0.8
	SE	2	2	2	2	1				
*Confectionery*	Mean	6 ^a^	11 ^b,c^	12 ^b^	14 ^c^	10	<0.0001	0.001	0.5	0.06
	SE	1	1	1	1	1				
*Sugar, jam and sweet spreads*	Mean	12 ^a,c^	9 ^b^	13 ^a^	9 ^b,c^	11	<0.0001	0.003	0.001	0.9
	SE	1	1	1	1	1				

Values sharing the same postscript or none are not significantly different (multiple comparison *t*-test with Bonferroni correction). Italics denote food subgroups. Planned contrasts of LCB *vs.* each of the other beverages as specified a priori. Tests assume equal/unequal variance based on Levene’s test. No significant differences in consumption of fats, cheese, eggs.

### 3.4. Energy and Macronutrient Intake

**Figure 1:**
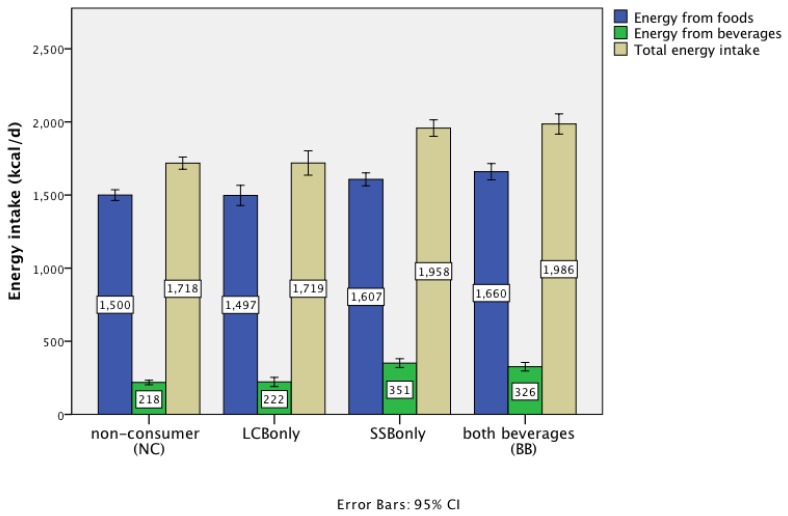
Energy intake from food and beverage sources according to beverage consumer group.

Adults consuming LCB had a mean total energy intake (TEI) identical to NC (1719, SE 21 *vs.* 1718 kcal/day, SE 42) and significantly lower than SSB consumers (1958 kcal/day SE 29), or BB (1986 kcal/day, SE 35) (Table 5), with a mean difference of 239 kcal, SE 51 between LCB consumers and SSB consumers (*p* < 0.0001). NC and LCB consumers had lower energy intakes from food as well as from beverages, compared to SSB consumers and consumers of both types (Figure 1). Overall, approximately 40%–50% of the total energy difference between groups was attributable to foods rather than beverages. NC and LCB consumers had significantly lower intakes of (non-milk extrinsic) sugars (both as g/day and % energy) compared with SSB and BB consumers (*p* < 0.0001) (Table 5). Intakes of fat and saturated fatty acids (SFA) were lower on an absolute basis (g/day) (*p* < 0.01) but not as a percentage of energy. Protein intakes were higher (as a percentage of energy) in LCB consumers than any other group.

**Table 5:** Energy and macronutrient intake according to beverage consumer group.

Macronutrient Intake (per Day)		Beverage Consumer Group					Planned Contrasts			
		NC	LCB	SSB	BB	All	Overall ANOVA	LCB *vs.* NC	LCB *vs.* SSB	LCB *vs.* BB
*n*		598	216	476	300	1590				
Energy (kcal)	Mean	1718 ^a^	1719 ^a^	1958 ^b^	1986 ^b^	1844	<0.0001	1.0	<0.0001	<0.0001
	SE	21	42	29	35	15				
Protein (g)	Mean	72	74	75	77	74	0.1	0.4	0.9	0.3
	SE	1.2	1.9	1.0	1.4	0.7				
Fat (g)	Mean	66 ^a^	65 ^a^	72 ^b^	74 ^b^	69	<0.0001	0.6	0.001	<0.0001
	SE	1.0	2.0	1.2	1.6	0.7				
Carbohydrate (g)	Mean	200 ^a^	200 ^a^	239 ^b^	243 ^b^	221	<0.0001	1.0	<0.0001	<0.0001
	SE	2	5	3	4	2				
Saturated fatty acids (g)	Mean	24.4 ^a^	24.2 ^a^	26.7 ^b^	27.0 ^b^	25.6	<0.0001	0.8	0.01	0.007
	SE	0.4	0.8	0.5	0.6	0.3				
Monounsaturated fatty acids (g)	Mean	23.2 ^a^	22.9 ^a^	26.0 ^b^	27.1 ^b^	24.8	<0.0001	0.7	<0.0001	<0.0001
	SE	0.4	0.7	0.5	0.6	0.3				
*n*-6 Fatty acids (g)	Mean	9.5 ^a^	9.2 ^a^	10.2 ^a,b^	10.6 ^b^	9.9	<0.0001	0.4	0.009	0.001
	SE	0.2	0.3	0.2	0.3	0.1				
*n*-3 Fatty acids (g)	Mean	2.1	1.9	2.1	2.1	2.1	0.096	0.02	0.05	0.02
	SE	0.1	0.1	0.1	0.1	0.03				
Trans fatty acids (g)	Mean	1.34 ^a^	1.37 ^a,b^	1.48 ^b^	1.49 ^b,c^	1.42	0.004	0.7	0.1	0.1
	SE	0.03	0.06	0.03	0.04	0.02				
Starch (g)	Mean	117 ^a^	118 ^a^	128 ^b^	138 ^c^	125	<0.0001	0.9	0.007	<0.0001
	SE	2	3	2	3	1				
Non-milk extrinsic sugars (g)	Mean	43 ^a^	43 ^a^	76 ^b^	71 ^b^	59	<0.0001	1.0	<0.0001	<0.0001
	SE	1	2	2	2	1				
Non-starch polysaccharide (g)	Mean	13.9	13.9	13.3	13.3	13.6	0.200	1.0	0.3	0.2
	SE	0.2	0.4	0.2	0.3	0.1				
**Macronutrients (% energy)**										
Protein	Mean	17.1 ^a^	18 ^b^	15.7 ^c^	15.7 ^c^	16.5	<0.0001	0.03	<0.0001	<0.0001
	SE	0.2	0.4	0.2	0.2	0.1				
Fat	Mean	33.9	33.2	33.0	33.2	33.4	0.1	0.3	0.6	0.9
	SE	0.3	0.5	0.3	0.3	0.2				
Carbohydrate	Mean	44.5 ^a^	44.1 ^a^	46.4 ^b^	46.4 ^b^	45.4	<0.0001	0.5	<0.0001	0.001
	SE	0.2	0.5	0.3	0.4	0.2				
Saturated fatty acids	Mean	12.6	12.4	12.2	12.1	12.4	0.1	0.4	0.6	0.4
	SE	0.4	0.8	0.5	0.7	0.3				
Monounsaturated fatty acids	Mean	11.9	11.8	11.9	12.1	11.9	0.5	0.6	0.6	0.1
	SE	0.4	0.7	0.5	0.6	0.3				
*n*-3 Fatty acids	Mean	1.1 ^a^	1.0 ^b^	0.9 ^b^	1.0 ^b^	1.0	<0.0001	0.005	0.6	0.9
	SE	0.05	0.07	0.05	0.06	0.03				
*n*-6 Fatty acids	Mean	4.9	4.8	4.7	4.8	4.8	0.2	0.3	0.6	1.0
	SE	0.19	0.31	0.19	0.25	0.11				
Trans fatty acids	Mean	0.7	0.7	0.7	0.7	0.7	0.4	0.6	0.8	0.5
	SE	0.03	0.06	0.03	0.04	0.02				
Starch	Mean	26.2 ^a^	26.2 ^a,b^	25.0 ^b^	26.4 ^a^	25.9	0.003	1.0	0.03	0.6
	SE	0.3	0.5	0.3	0.3	0.1				
Non-milk extrinsic sugars	Mean	9.4 ^a^	9.2 ^a^	14.3 ^b^	13.3 ^b^	11.6	<0.0001	0.6	<0.0001	<0.0001
	SE	0.2	0.3	0.2	0.3	0.2				

Values sharing the same postscript or none are not significantly different (multiple comparison *t*-test with Bonferroni correction).

### 3.5. Micronutrient Intakes

Micronutrient intakes were mostly similar across beverage groups, although NC had lower mean sodium intake and higher vitamin A and D intakes compared with BB consumers (Table 6). LCB consumers had mean intakes intermediate between NC and SSB and not significantly different from either.

**Table 6:** Mean micronutrient intake and contrasts according to beverage consumption group.

Micronutrient (per Day)		Beverage Consumer Group					Planned Contrasts			
		NC	LCB	SSB	BB	All	Overall	LCB *vs.* non	LCB *vs.* SSB	LCB *vs.* BB
	*n*	598	216	476	300	1590				
Vitamin A (RE) (µg)	Mean	1344 ^a^	1119 ^a,b^	1012 ^b^	976 ^b,c^	1143	<0.0001	0.04	0.3	0.2
	SE	56	91	40	58	29				
Thiamin (mg)	Mean	2.3	1.9	1.6	2.2	2.0	0.2	0.2	0.2	0.5
	SE	0.3	0.2	0.1	0.4	0.2				
Riboflavin (mg)	Mean	2.2	1.8	1.8	2.1	2.0	0.3	0.07	0.9	0.3
	SE	0.2	0.1	0.1	0.3	0.1				
Niacin equivalent (mg)	Mean	37.0	38.0	39.1	39.7	38.3	0.09	0.5	0.4	0.2
	SE	0.8	1.1	0.7	0.9	0.4				
Vitamin B6 (mg)	Mean	2.8	2.6	2.6	3.2	2.8	0.2	0.4	0.9	0.1
	SE	0.2	0.1	0.1	0.4	0.1				
Vitamin B12 (µg)	Mean	6.6 ^a^	5.7 ^a,b^	5.5 ^b^	5.7 ^a,b^	6.0	0.02	0.05	0.5	1.0
	SE	0.3	0.3	0.2	0.4	0.2				
Folate (µg)	Mean	301.1	287.4	279.7	300.8	292.6	0.5	0.4	0.5	0.6
	SE	10.9	10.5	6.1	21.8	6.2				
Vitamin C (mg)	Mean	106.6	99.0	108.9	101.2	105.4	0.7	0.4	0.3	0.8
	SE	5.8	6.7	5.9	5.3	3.1				
Vitamin D (µg)	Mean	4.4 ^a^	3.9 ^a,b^	3.2 ^b^	3.4 ^b,c^	3.8	<0.0001	0.1	0.02	0.07
	SE	0.2	0.3	0.1	0.2	0.1				
Vitamin E (mg)	Mean	12.5	10.5	11.4	10.9	11.6	0.5	0.08	0.4	0.7
	SE	1.0	0.6	0.9	1.0	0.5				
Iron (mg)	Mean	11.8	11.5	12.2	11.6	11.9	0.7	0.7	0.3	0.9
	SE	0.3	0.5	0.6	0.3	0.2				
Calcium (mg)	Mean	824	842	851	866	842	0.3	0.5	0.8	0.4
	SE	13	24	15	20	9				
Magnesium (mg)	Mean	258	256	259	254	257	0.9	0.8	0.8	0.8
	SE	4	7	4	5	2				
Potassium (mg)	Mean	2806	2809	2775	2786	2793	0.9	1.0	0.7	0.8
	SE	36	71	40	51	22				
Zinc (mg)	Mean	9.5	9.6	9.5	9.8	9.6	0.8	0.8	0.7	0.7
	SE	0.2	0.4	0.2	0.3	0.1				
Selenium (µg)	Mean	51	50	48	49	50	0.5	0.9	0.3	0.6
	SE	1.3	2.0	1.0	1.4	0.7				
Iodine (µg)	Mean	180 ^a^	176 ^a,b^	173 ^a,b^	160 ^b^	174	0.02	0.6	0.7	0.05
	SE	3.6	6.7	4.0	5.0	2.2				
Sodium (mg)	Mean	2112 ^a^	2244 ^a,b^	2355 ^b^	2559 ^c^	2288	<0.0001	0.06	0.1	<0.0001
	SE	32	62	40	50	22				

Values sharing the same postscript or none are not significantly different (multiple comparison *t*-test with Bonferroni correction).

### 3.6. Energy Intake: Adjustment for Covariates

Lower energy intakes in LCB and NC (compared with SSB and BB consumers) remained significant after adjustment for sex, age group, ethnicity, BMI status, dieting, smoking, education, social class and income (Table 7). There were no significant interactions. Adjusted means for energy intake were 1620, 1604, 1794, 1846 kcal/day in NC, LCB, SSB and BB consumers, respectively, with a difference of 190 kcal between LCB consumers and SSB consumers (*p* < 0.001; Pairwise comparison test with Bonferroni correction).

**Table 7:** Regression model of beverages on total energy intake, adjusted for covariates.

Parameter	B	Std. Error	t	Sig.
Intercept	1502	93	16	<0.0001
Non Consumer	−226	43	−5	<0.0001
Low Calorie Beverages	−241	52	−5	<0.0001
Sugar-Sweetened Beverages	−52	42	−1	0.2
Both Beverages (ref)	0			
Males	515	29	18	<0.0001
Females (ref)	0			
Age 16–24 years	9	59	0	0.9
Age 25–44 years	56	48	1	0.2
Age 45–64 years	22	45	0	0.6
Age 65 + years (ref)	0			
BMI < 18.5 (underweight)	95	110	1	0.4
BMI 18.5 < 25 (normal weight)	58	38	1	0.1
BMI 25 < 30 (overweight)	18	37	0	0.6
BMI 30+ (obese) (ref)	0			
Not dieting	121	46	3	0.008
Dieting (ref)	0			
White ethnicity	161	49	3	0.001
Non-white ethnicity (ref)	0			
Current smoker	−91	38	−2	0.02
Ex-smoker	10	37	0	0.8
Non-smoker (ref)	0	.	.	.
Socioeconomic group 1 (highest)	77	48	2	0.1
Socioeconomic group 2	6	38	0	0.9
Socioeconomic group 3	16	41	0	0.7
Socioeconomic group 4 (lowest) (ref)	0			
Household income 1 (lowest)	−101	54	−2	0.06
Household income 2	−46	44	−1	0.3
Household income 3	−85	41	−2	0.04
Household income 4 (highest) (ref)	0			
Educational qualification (none)	−118	42	−3	0.006
Educational qualification (any) (ref)	0			

Univariate GLM using weighted least squares. B (beta) coefficients are the difference in total energy intake associated with belonging to each group compared to the reference, after adjustment for all other factors in the model. There were no significant interactions. Adjusted difference between LCB and SSB = 190 kcal (SE 48) *p* = 0.001 with Bonferroni adjustment for multiple comparison.

### 3.7. Within- Person Analysis

Table 8 shows the coefficients estimating the impact of consuming 100 g of each beverage on total energy intake (7.1) energy from beverages (7.2) and food energy (7.3) if all other beverages and foods are allowed to vary (as they do in practice). Each 100 g of SSB was associated with an energy difference of +41 kcal (95% CI 33, 49); coefficients for other energetic beverages (milk, fruit juice, alcoholic beverages) ranged from +31 to +51 kcal. Consumption of LCB was not associated with any significant change in energy intake (0.6 kcal/100 g (*p* = 0.89), neither was the coefficient for tea, coffee and water significantly different from zero (−3 kcal/100 g; *p* = 0.073). By comparison the energy density of beverages (kcal/100 g based on total volume consumed) were as follows: LCB = 1; tea/coffee/water ≤1; SSB = 33; FJ = 37; milk = 52; alcoholic beverages = 48.

In Table 9, models 8.1, 8.2, 8.3 are substitution models of the effect on TEI, beverage energy and food energy, respectively, when replacing LCB with each of the other beverages, whilst keeping other beverage amounts constant. The coefficients can be compared with the true energy density of the beverages to estimate compensation, whether positive or negative. Consuming LCB instead of SSB (with other beverages constant) was associated with a 39 kcal reduction in total energy per 100 g substituted (8.1: TEI). In 8.2 (beverage energy) the coefficient for SSB (32 kcal/100 g) was similar to the actual energy density of all SSB consumed (33 kcal/100 g), suggesting weak or no compensation in beverage energy. 8.3 shows that food energy increased slightly by 7 kcal/100 g when consuming SSB instead of LCB (or food intake was 7 kcal lower when LCB was consumed); however this was not significant (*p* = 0.15).

This analysis suggests that LCB has no independent association with energy or food intake. Consuming more LCB on any one day does not appear to be associated with a lower energy intake that day. Strict 1:1 substitution of LCB in place of SSB could save an estimated 32 kcal/100 g (beverage energy model) assuming no change in intake of food or other beverages. This is equivalent to 106 kcal for a standard can of soda (330 g).

**Table 8:** Within-person regression models of the impact of each beverage on total energy, beverage energy and food energy intake (kcal).

Model 7.1				Model 7.2				Model 7.3			
Total Energy				Beverage Energy				Food Energy			
Parameter	B	SE	*p* Value	Parameter	B	SE	*p* Value	Parameter	B	SE	*p* Value
Low Calorie Beverages	1	4	0.9	Low Calorie Beverages	−5	2	0.01	Low Calorie Beverages	6	4	0.1
Sugar-Sweetened Beverages	41	4	<0.0001	Sugar-Sweetened Beverages	28	2	<0.0001	Sugar-Sweetened Beverages	14	3	<0.0001
Tea/coffee/water	−3	2	0.07	Tea/coffee/water	−9	1	<0.0001	Tea/coffee/water	6	2	<0.0001
Milk	51	7	<0.0001	Milk	39	3	<0.0001	Milk	11	6	0.05
Fruit juice	31	7	<0.0001	Fruit juice	21	3	<0.0001	Fruit juice	10	6	0.09
Alcoholic beverages	37	1	<0.0001	Alcoholic beverages	38	0	<0.0001	Alcoholic beverages	−1	1	0.2

Combined results of separate models for each beverage. Coefficients (B) represent the simple (crude) effect of 100 g of beverage on energy intake (in kcals). Amounts of other beverages were allowed to vary. Models were adjusted for day of week and day number.

**Table 9:** Within-person regression models of the impact of substituting beverages for Low Calorie Beverages.

8.1. Total Energy				8.2. Beverage Energy				8.3. Food Energy			
(Adjusted R Squared = 0.638)			*p* Value	(Adjusted *R* Squared = 0.980)			*p* Value	(Adjusted *R* Squared = 0.507)			*p* Value
Parameter	B	SE		Parameter	B	SE		Parameter	B	SE	
Intercept	1440	222	0.0001	Intercept	−89	52	0.09	Intercept	1522	215	0.0001
Low Calorie Beverages *	-			Low Calorie Beverages *	-			Low Calorie Beverages *	-		
Sugar-Sweetened Beverages	39	5	0.0001	Sugar-Sweetened Beverages	32	1	0.0001	Sugar-Sweetened Beverages	7	5	0.2
Tea/coffee/water	−9	4	0.03	Tea/coffee/water	−5	1	0.0001	Tea/coffee/water	−3	4	0.4
Milk	54	7	0.0001	Milk	52	2	0.0001	Milk	3	7	0.7
Fruit juice	38	7	0.0001	Fruit juice	33	2	0.0001	Fruit juice	5	7	0.5
Alcoholic beverages	24	4	0.0001	Alcoholic beverages	35	1	0.0001	Alcoholic beverages	−11	4	0.006
ALL BEVERAGES	16	4	0.0001	ALL BEVERAGES	5	1	0.0001	ALL BEVERAGES	11	4	0.004
Friday	67	23	0.004	Friday	13	5	0.02	Friday	54	23	0.02
Saturday	134	24	0.0001	Saturday	26	6	0	Saturday	108	24	0.0001
Sunday	39	25	0.1	Sunday	1	6	0.9	Sunday	37	24	0.1
Monday	−18	25	0.5	Monday	−3	6	0.6	Monday	−14	24	0.5
Tuesday	−3	24	0.9	Tuesday	1	6	0.9	Tuesday	−4	23	0.9
Wednesday (ref)	0			Wednesday (ref)	0			Wednesday (ref)	0		
Thursday	19	23	0.4	Thursday	−2	5	0.8	Thursday	20	23	0.4
Day1	−10	16	0.5	Day1	−3	4	0.5	Day1	−8	16	0.6
Day2	5	16	0.8	Day2	0	4	1.00	Day2	5	15	0.8
Day3	16	16	0.3	Day3	2	4	0.7	Day3	15	15	0.3
Day4 (ref)	0			Day4 (ref)	0			Day4 (ref)	0		

* Models show estimated caloric changes on consuming 100 g each beverage type instead of 100 g LCB whilst keeping total beverage intake constant (reverse sign applies for substituting LCB in place of each beverage).

## 4. Discussion

Our findings do not support the assertion that LCB negatively affect diet because adults who drink LCB compensate by eating more sugary or fatty foods [26,27]. Sugar intakes were lower among LCB consumers than SSB consumers owing mainly to the contribution from beverages in the SSB consumers, and there was no evidence of a reciprocal relationship with fat (or “sugar: fat see-saw”) [28] between LCB and SSB consumer groups; fat intakes were lower in LCB consumers on a g/day basis, and percentage energy from fat was not significantly different. Protein intakes (as a percentage of energy) were higher among LCB consumers than other groups, and the diets of NC and LCB consumers contained similar amounts of micronutrients for fewer calories than other groups. In all main respects (energy, macronutrient and micronutrient intakes), the diets of the LCB group were similar to those who consumed no soft drinks at all (NC), except that total fluid intakes were lower in NC. Consumption of fruit, vegetables and fish declined across the groups (NC > LCB > SSB >BB) with the reverse trend for meat; however not all differences were statistically significant. NC were significantly older than LCB and SSB consumers, while BB consumers were younger. Hence some of the observed differences may be age-related; for example older people are more likely to achieve recommendations for fruit and vegetable consumption [1].

Our conclusions are similar to those of Drewnowski and Rehm, who reported higher healthy eating index scores among users of low calorie sweeteners (LCS) and LCB compared with non-consumers, as well as healthier behaviours such as not smoking and taking more exercise [23]. The counter-intuitive observation in both studies that LCB users are more likely to be overweight despite a lower reported EI than non-users is most likely attributable to reverse causality [29]. Unlike Drewnowski and Rehm we did not find LCB consumers to have higher intakes of saturated fat and sodium. Methodological differences between our studies may explain this: LCB users may include some participants who consumed SSB in addition to LCB (our BB group), and LCB non-users may include those who consume neither LCB nor soft drinks (our NC group), whereas our classification discriminated on both classes of beverage.

Findings contrast with those of Piernas *et al.* who reported that overall diet quality was lower in LCB-only consumers as well as in SSB consumers, compared with non/low consumers [30] and also that purchases of either LCB or SSB were associated with more energy from food, more sugar and fat and more desserts [27]. An acknowledged limitation of purchase studies is the indirect measure of consumption and omission of unpackaged foods and out-of-home eating occasions. Furthermore, stronger evidence (from the CHOICE randomised control trial) indicated that those who replaced caloric drinks with either LCB or water also reduced their consumption of added sugar and desserts, with the LCB group sustaining a larger reduction in desserts than the water group [31].

Our within-person results show some similarities with other studies using multiple records in American populations. Wang *et al.* reported no significant association between LCB intake and total energy in children when LCB was added to the diet [32]. In models replacing SSB they only found a significant energy reduction with plain water and not LCB, however low numbers of children consuming diet drinks may have limited the statistical power. Stookey *et al.*, in a study of 118 overweight women followed up for 12 months, reported that replacing SSBs by LCBs was associated with a reduction in energy intake but this was 30% smaller than if replaced by water [33]. Finally in a recent study using within-person data (2 days) from NHANES, consuming SSB was associated with an energy increment of 226 kcal/day, compared with 69 kcal associated with LCB (net difference of 157 kcal/day) [34]. Although there was a small increase in food calories this did not negate the energy saving associated with consuming LCB instead of SSB. From within-person analysis of NDNS, we found that substitution of SSB by LCB or by water/tea/coffee was associated with a reduction in total energy, and this was attributable to lower energy from beverages, with no evidence of increased food energy.

### Strengths and Limitations of the Present Study

Most work on the effects of beverages on intakes and on health focuses on the adverse impacts of SSB, while less has addressed potential benefits of LCB. A major strength of this study is the NDNS data, which is nationally representative of the UK and of high quality. The 4-day diary provides a better representation of usual consumption than the more common 24-h dietary recall. Four days of records also allowed us to estimate the impact of dietary change on energy intake whilst controlling for inter-personal differences.

Causality cannot be inferred from the associations found in this analysis due to its cross-sectional design. However, the results are consistent with other data suggesting that beverages tend to supplement food choices in an independent manner, so that the benefit of LCB in terms of energy and sugars intake derives from substitution of SSB. Misreporting is a known weakness of self–reported diet records [35] and unfortunately there is no reliable means of correcting for this or reliably identifying individuals who misreport [36]. However, our conclusions regarding lower NMES intakes are based on energy-adjusted values, less susceptible to misreporting effects [37,38]. In energy intake regressions we included an adjustment for dieting, smoking, BMI and other covariates often associated with energy intake or underreporting, although residual confounding can never be ruled out. The within-person analysis further reduces distortions caused by mis-reporting because individuals tend to be consistent in this regard [39].

## 5. Conclusions

LCB provide a palatable source of water with minimal sugar and energy content. Their caloric benefits derive from their role as substitutes for SSB and meta-analyses have demonstrated that replacing SSB with LCB leads to reduced energy intake and modest weight loss [18]. Maintaining good diet quality during weight loss is important in order to meet nutrient requirements at a lower energy intake. In UK adults we found that LCB consumers and NC consumed less energy and sugars than consumers of SSB, or both types. NC and LCB consumers tended to have higher quality diets compared with SSB and BB consumers and did not compensate for the sugar or energy deficit by consuming more sugary foods.
